# Tick mitochondrial genomes: structural characteristics and phylogenetic implications

**DOI:** 10.1186/s13071-019-3705-3

**Published:** 2019-09-13

**Authors:** Tianhong Wang, Shiqi Zhang, Tingwei Pei, Zhijun Yu, Jingze Liu

**Affiliations:** Hebei Key Laboratory of Animal Physiology, Biochemistry and Molecular Biology, College of Life Sciences, Hebei Normal University, Shijiazhuang, 050024 China

**Keywords:** Ticks, Mitochondrial genome (mt-genome), Gene structure, Phylogeny

## Abstract

Ticks are obligate blood-sucking arachnid ectoparasites from the order Acarina, and many are notorious as vectors of a wide variety of zoonotic pathogens. However, the systematics of ticks in several genera is still controversial. The mitochondrial genome (mt-genome) has been widely used in arthropod phylogeny, molecular evolution and population genetics. With the development of sequencing technologies, an increasing number of tick mt-genomes have been sequenced and annotated. To date, 63 complete tick mt-genomes are available in the NCBI database, and these genomes have become an increasingly important genetic resource and source of molecular markers in phylogenetic studies of ticks in recent years. The present review summarizes all available complete mt-genomes of ticks in the NCBI database and analyses their characteristics, including structure, base composition and gene arrangement. Furthermore, a phylogenetic tree was constructed using mitochondrial protein-coding genes (PCGs) and ribosomal RNA (rRNA) genes from ticks. The results will provide important clues for deciphering new tick mt-genomes and establish a foundation for subsequent taxonomic research.

## Background

Ticks are obligate blood-sucking arachnid ectoparasites that can feed on a wide range of vertebrates, including mammals, birds and reptiles [1, 2]. Ticks are well-known zoonotic pathogen vectors, and tick-borne diseases (TBDs) are increasingly threatening animal and human health, thereby causing great economic damage [3, 4]. Many important tick-borne pathogens have been characterized from ticks in recent years, including *Anaplasma bovis*, *Babesia ovata*, *Rickettsia japonica*, *Chlamydiaceae* bacteria and severe fever with thrombocytopenia syndrome virus (SFTSV), which have attracted increasing attention in the field of public health [5–9]. Recently, a newly segmented virus with a febrile illness similar in its clinical manifestation to tick-borne encephalitis virus (TBEV) was discovered, which was designated as Alongshan virus (ALSV) and confirmed in 86 patients from several provinces in China [10]. Globally, the annual financial losses due to ticks and TBDs are in the billions of dollars [3, 11]. A total of 896 tick species have been described worldwide in three families: Ixodidae (hard ticks, 702 species), Argasidae (soft ticks, 193 species) and Nuttalliellidae (1 species) [12–14]. Hard ticks possess a sclerotized scutum in all life stages except eggs, have an apically located gnathostoma, usually feed for several days and ingest a large amount of blood [15, 16]. Soft ticks have no sclerotized scutum and mouthparts located anteroventrally. The ticks usually feed and expand the body within minutes to hours [17]. *Nuttalliella namaqua* is the unique species in the family Nuttalliellidae, and it displays many characteristics associated with hard and soft ticks and can engorge as rapidly as soft ticks [18]. The differences in life history, behaviour, and morphological characteristics are useful for the discrimination of soft ticks and hard ticks, but there are still numerous difficulties among the interspecies taxonomic characterization and geographical origin of ticks, especially for soft ticks [19]. Therefore, the increasing number of characterized mt-genomes has shown considerable potential in tick phylogeny, molecular evolution and population genetics.

The mt-genome is characterized by low molecular weight, high copy quantity and genetic conservation. The mt-genome has been widely used in molecular evolution, phylogeny and genealogy in recent years [20–22]. Similar to other arthropods, the tick mt-genome has a circular, double-stranded DNA structure with a length of 14–16 kb and a total of 37 genes, including 13 protein-coding genes, 22 transfer RNA genes (tRNAs) and 2 rRNA genes [20, 23]. With the development of next-generation sequencing (NGS) technology, increasing numbers of complete mt-genomes have been sequenced and annotated from various tick species [24]. The complete mt-genome sequences are necessary for advances in areas that are crucial for TBDs study and control [24]. To date, 63 complete tick mt-genomes are available in the NCBI database, and these genomes have become an increasingly important genetic resource and source of molecular markers in phylogenetic studies of ticks in recent years [19, 25]. Hence, in the present study, we used the MITOS online software (http://mitos.bioinf.uni-leipzig.de/index.py/) to annotate the complete mt-genomes of ticks and compare their characteristics, including structure, base composition and gene arrangement. Furthermore, a phylogenetic tree was constructed using PCGs and rRNA genes from ticks. The results will provide important clues for deciphering new tick mt-genomes and provide insights for subsequent taxonomic research.

### Present state of research on tick mt-genomes

The first mt-genomes of ticks (*Ixodes hexagonus* and *Rhipicephalus sanguineus*) were reported by Black et al. [26] in 1998. As of May 2019, 63 complete tick mt-genomes have been deposited in the NCBI database. Most tick mt-genomes were published in this decade, and are from 3 families and 15 genera, including 35 species in the family Ixodidae: *Ixodes* (7 species); *Amblyomma* (7 species); *Rhipicephalus* (5 species); *Rhipicentor* (1 species); *Dermacentor* (4 species); *Bothriocroton* (2 species); *Haemaphysalis* (8 species); and *Hyalomma* (1 species) [26–41]; 27 species in the family Argasidae: *Argas* (8 species); *Antricola* (1 species); *Carios* (2 species); *Ornithodoros* (14 species); *Otobius* (1 species); and *Nothoaspis* (1 species) [19, 27, 42–44]; and 1 *Nuttalliella* species in family Nuttalliellidae [44] (Table 1). In recent years, phylogenetic studies based on mt-genome sequences have been effectively carried out for many tick species [21, 28–30, 36, 40]. These achievements are also essential for understanding the genetic differentiation and phylogeny of ticks [31–34]. However, the genera *Anomalohimalaya*, *Compluriscutula*, *Margaropus* and *Nosomma* still lack complete mt-genome information, and most species were sampled in a limited geographical area [45]. Complete mt-genome sequences have only been obtained for approximately 7% (63/896) of the tick species, and the general characteristics of most tick mt-genomes remain to be determined.

**Table 1:** The available tick complete mitochondrial genomes in GenBank

Family	Genus	Species	GenBank ID	Reference
Nuttalliellidae	*Nuttalliella*	*N. namaqua*	JQ665719	Mans et al. [44]
Argasidae	*Argas*	*A. africolumbae*	KJ133580	Mans et al. [44]
		*A. boueti*	KR907234	Mans et al. [Unpublished]^a^
		*A. brumpti*	KR907226	Mans et al. [Unpublished]
		*A. lagenoplastis*	KC769587	Burger et al. [27]
		*A. miniatus*	KC769590	Burger et al. [27]
		*A. persicus*	KJ133581	Mans et al. [Unpublished]
		*A. striatus*	KJ133583	Mans et al. [Unpublished]
		*A. walkerae*	KJ133585	Mans et al. [Unpublished]
	*Antricola*	*A. mexicanus*	KC769591	Burger et al. [27]
	*Carios*	*C. capensis*	AB075953	Fukunaga et al. [Unpublished]
		*C. faini*	KJ133589	Mans et al. [Unpublished]
	*Nothoaspis*	*N. amazoniensis*	KX712088	Lima et al. [Unpublished]
	*Ornithodoros*	*O. brasiliensis*	KC769593	Burger et al. [27]
		*O. compactus*	KJ133590	Mans et al. [Unpublished]
		*O. coriaceus*	MG593161	Mans et al. [Unpublished]
		*O. costalis*	KJ133591	Mans et al. [Unpublished]
		*O. hermsi*	MF818032	Mans et al. [Unpublished]
		*O. moubata*	AB073679	Fukunaga et al. [43]
		*O. parkeri*	MF818029	Mans et al. [Unpublished]
		*O. porcinus*	AB105451	Mitani et al. [42]
		*O. rostratus*	KC769592	Burger et al. [27]
		*O. savignyi*	KJ133604	Mans et al. [Unpublished]
		*O. sonrai*	MF818026	Mans et al. [Unpublished]
		*O. tholozani*	MF818023	Mans et al. [Unpublished]
		*O. turicata*	MF818021	Mans et al. [Unpublished]
		*O. zumpti*	KR907257	Mans et al. [Unpublished]
	*Otobius*	*O. megnini*	KC769589	Burger et al. [27]
Ixodidae	*Ixodes*	*I. hexagonus*	AF081828	Black et al. [26]
		*I. holocyclus*	AB075955	Shao et al. [41]
		*I. pavlovskyi*	KJ000060	Mikryukova et al. [Unpublished]
		*I. persulcatus*	KU935457	Sui et al. [40]
		*I. ricinus*	JN248424	Montagna et al. [39]
		*I. tasmani*	MH043269	Burnard et al. [25]
		*I. uriae*	AB087746	Shao et al. [37]
	*Amblyomma*	*A. americanum*	KP941755	Williams-Newkirk et al. [36]
		*A. cajennense*	JX573118	Burger et al. [29]
		*A. elaphense*	JN863729	Burger et al. [29]
		*A. fimbriatum*	JN863730	Burger et al. [28]
		*A. sculptum*	KX622791	Lima et al. [31]
		*A. sphenodonti*	JN863731	Burger et al. [29]
		*A. triguttatum*	AB113317	Fukunaga et al. [Unpublished]
	*Rhipicephalus*	*R. australis*	KC503255	Burger et al. [27]
		*R. geigyi*	KC503263	Burger et al. [27]
		*R. microplus*	KC503261	Burger et al. [30]
		*R. sanguineus*	JX416325	Liu et al. [32]
		*R. turanicus*	KY996841	Li et al. [Unpublished]
	*Rhipicentor*	*R. nuttalli*	MF818020	Mans et al. [Unpublished]
	*Dermacentor*	*D. verestianus*	MG986896	Yu et al. [35]
		*D. nitens*	KC503258	Burger et al. [27]
		*D. nuttalli*	KT764942	Guo et al. [33]
		*D. silvarum*	KP258209	Chang et al. [Unpublished]
	*Bothriocroton*	*B. concolor*	JN863727	Burger et al. [28]
		*B. undatum*	JN863728	Burger et al. [28]
	*Haemaphysalis*	*H. bancrofti*	MH043268	Burnard et al. [25]
		*H. concinna*	KY364906	Fu et al. [38]
		*H. flava*	AB075954	Shao et al. [41]
		*H. formosensis*	JX573135	Burger et al. [29]
		*H. hystricis*	MH510034	Tian et al. [Unpublished]
		*H. japonica*	MG253031	Fu et al. [Unpublished]
		*H. longicornis*	MG450553	Geng et al. [Unpublished]
		*H. parva*	JX573136	Burger et al. [29]
	*Hyalomma*	*H. asiaticum*	MF101817	Liu et al. [34]

^a^Unpublished here refers to the sequences deposited into GenBank only without paper published

### Basic features of tick mt-genomes

The length of the mt-genomes of ticks average 14,633 bp, with the longest reaching 15,227 bp (*Ixodes tasmani*) and the smallest measuring only 14,307 bp (*Argas boueti*) (Table 2). Generally, the length of the mt-genomes from hard ticks is slightly longer than that of soft ticks (14,796 and 14,429 bp, respectively). The length differences of the mt-genomes between ticks may be influenced by gene rearrangement and the length of the non-coding regions (NCRs) [46, 47]. MITOS online analysis showed no gene deletion or duplication in tick mt-genomes, which contain 13 PCGs, 2 rRNA genes and 22 tRNA genes. Among the 13 PCGs, 9 PCGs (*nad*2, *cox*1, *cox*2, *atp*8, *atp*6, *cox*3, *nad*3, *nad*6, *cytb*) are located in the majority strand (J strand) and 4 PCGs (*nad*5, *nad*4, *nad*4*L*, *nad*1) are located in the minority strand (N strand).

**Table 2:** The base features of tick mitochondrial genomes

Species	Mitochondrial genome base content								PCGs base content							
	Length	A + T (%)	A	T	AT-skew	G	C	GC-skew	Length	A + T (%)	A	T	AT-skew	G	C	GC-skew
*Nuttalliella namaqua*	14,425	78.59	5864	5472	0.035	1097	1992	− 0.290	10,792	78.64	3756	4731	− 0.115	1150	1155	− 0.002
*Argas africolumbae*	14,440	73.35	5579	5013	0.053	1311	2537	− 0.319	10,951	72.64	3327	4628	− 0.164	1408	1588	− 0.060
*Argas boueti*	14,307	76.63	5768	5196	0.052	1152	2191	− 0.311	10,830	76.24	3660	4597	− 0.113	1214	1359	− 0.056
*Argas brumpti*	14,516	69.91	5094	5054	0.004	1326	3042	− 0.393	10,834	68.42	2926	4487	− 0.211	1571	1850	− 0.082
*Argas lagenoplastis*	14,478	72.64	5594	4923	0.064	1340	2621	− 0.323	10,864	71.76	3267	4529	− 0.162	1478	1590	− 0.037
*Argas miniatus*	14,416	74.16	5452	5239	0.020	1252	2473	− 0.328	10,820	73.56	3248	4711	− 0.184	1428	1433	− 0.002
*Argas persicus*	14,411	72.72	5427	5053	0.036	1264	2667	− 0.357	10,866	71.83	3217	4588	− 0.176	1502	1559	− 0.019
*Argas striatus*	14,485	76.22	5739	5302	0.040	1167	2277	− 0.322	10,844	75.89	3455	4774	− 0.160	1266	1349	− 0.032
*Argas walkerae*	14,437	74.36	5488	5247	0.022	1213	2489	− 0.345	10,865	73.65	3313	4689	− 0.172	1377	1486	− 0.038
*Antricola mexicanus*	14,415	74.60	5706	5047	0.061	1242	2418	− 0.321	10,813	73.80	3547	4433	− 0.111	1422	1410	0.004
*Carios capensis*	14,418	73.54	5491	5112	0.036	1195	2620	− 0.374	10,875	72.66	3389	4513	− 0.142	1406	1567	− 0.054
*Carios faini*	14,433	76.68	5902	5165	0.067	1096	2270	− 0.349	10,883	75.97	3677	4591	− 0.111	1259	1356	− 0.037
*Ornithodoros brasiliensis*	14,489	73.16	5653	4947	0.067	1251	2638	− 0.357	10,843	72.24	3371	4462	− 0.139	1442	1568	− 0.042
*Ornithodoros compactus*	14,400	72.14	5530	4858	0.065	1265	2747	− 0.369	10,890	71.21	3335	4420	− 0.140	1557	1578	− 0.007
*Ornithodoros coriaceus*	14,423	69.75	5468	4592	0.087	1295	3068	− 0.406	10,917	67.90	3192	4221	− 0.139	1585	1919	− 0.095
*Ornithodoros costalis*	14,442	72.32	5343	5101	0.023	1285	2713	− 0.357	10,903	71.26	3277	4493	− 0.156	1460	1673	− 0.068
*Ornithodoros hermsi*	14,430	71.97	5368	5017	0.034	1348	2697	− 0.333	10,913	71.05	3306	4448	− 0.147	1520	1639	− 0.038
*Ornithodoros moubata*	14,398	72.26	5548	4856	0.067	1240	2754	− 0.379	10,885	71.36	3344	4423	− 0.139	1542	1576	− 0.011
*Ornithodoros parkeri*	14,437	74.45	5724	5024	0.065	1262	2427	− 0.316	10,868	73.94	3450	4586	− 0.141	1427	1405	0.008
*Ornithodoros porcinus*	14,378	70.98	5405	4801	0.059	1346	2826	− 0.355	10,876	70.11	3251	4374	− 0.147	1625	1626	0.000
*Ornithodoros rostratus*	14,452	72.96	5533	5011	0.050	1304	2604	− 0.333	10,836	72.16	3393	4426	− 0.132	1445	1572	− 0.042
*Ornithodoros savignyi*	14,401	65.23	5461	3933	0.163	1263	3744	− 0.496	10,889	63.59	3054	3870	− 0.118	1807	2158	− 0.089
*Ornithodoros sonrai*	14,430	74.02	5383	5298	0.008	1249	2500	− 0.334	10,866	73.23	3300	4657	− 0.171	1413	1496	− 0.029
*Ornithodoros tholozani*	14,407	69.34	5138	4852	0.029	1425	2992	− 0.355	10,880	67.87	3135	4249	− 0.151	1618	1878	− 0.074
*Ornithodoros turicata*	14,458	73.27	5653	4941	0.067	1325	2539	− 0.314	10,868	72.41	3398	4472	− 0.136	1461	1537	− 0.025
*Ornithodoros zumpti*	14,438	69.61	5063	4988	0.007	1452	2935	− 0.338	10,856	68.38	3129	4294	− 0.157	1635	1798	− 0.047
*Otobius megnini*	14,430	74.85	5609	5192	0.039	1172	2457	− 0.354	10,821	73.83	3408	4581	− 0.147	1355	1477	− 0.043
*Nothoaspis amazoniensis*	14,416	72.93	5671	4842	0.079	1172	2731	− 0.399	10,851	71.86	3488	4309	− 0.105	1447	1607	− 0.052
*Ixodes hexagonus*	14,539	72.66	5457	5107	0.033	1260	2715	− 0.366	10,826	71.13	3235	4465	− 0.160	1428	1698	− 0.086
*Ixodes holocyclus*	15,007	77.38	5728	5884	− 0.013	1266	2129	− 0.254	10,862	76.39	3524	4773	− 0.151	1305	1260	0.018
*Ixodes pavlovskyi*	14,575	78.09	5529	5852	− 0.028	1177	2017	− 0.263	10,888	77.24	3509	4901	− 0.166	1224	1254	− 0.012
*Ixodes persulcatus*	14,539	77.35	5496	5750	− 0.023	1202	2091	− 0.270	10,769	76.63	3456	4796	− 0.162	1217	1300	− 0.033
*Ixodes ricinus*	14,566	78.66	5594	5864	− 0.024	1147	1961	− 0.262	10,813	77.99	3537	4896	− 0.161	1155	1225	− 0.029
*Ixodes tasmani*	15,227	77.92	5936	5929	0.001	1200	2162	− 0.286	10,765	77.14	3549	4755	− 0.145	1207	1254	− 0.019
*Ixodes uriae*	15,053	74.79	5667	5591	0.007	1275	2520	− 0.328	10,837	73.75	3439	4553	− 0.139	1386	1459	− 0.026
*Amblyomma americanum*	14,709	76.78	5478	5816	− 0.030	1458	1957	− 0.146	10,811	76.68	3544	4746	− 0.145	1190	1331	− 0.056
*Amblyomma cajennense*	14,780	75.96	5444	5783	− 0.030	1488	2064	− 0.162	10,840	75.60	3468	4727	− 0.154	1251	1394	− 0.054
*Amblyomma elaphense*	14,627	80.45	5696	6072	− 0.032	1234	1625	− 0.137	10,815	80.46	3737	4965	− 0.141	1016	1097	− 0.038
*Amblyomma fimbriatum*	14,705	77.67	5601	5820	− 0.019	1385	1899	− 0.157	10,874	77.19	3600	4794	− 0.142	1155	1325	− 0.069
*Amblyomma sculptum*	14,780	76.10	5454	5794	− 0.030	1482	2050	− 0.161	10,840	75.80	3477	4740	− 0.154	1243	1380	− 0.052
*Amblyomma sphenodonti*	14,772	77.78	5585	5905	− 0.028	1438	1844	− 0.124	10,874	77.67	3595	4851	− 0.149	1169	1259	− 0.037
*Amblyomma triguttatum*	14,740	78.40	5653	5903	− 0.022	1381	1803	− 0.133	10,876	78.29	3607	4908	− 0.153	1098	1263	− 0.070
*Rhipicephalus australis*	14,891	79.89	5789	6108	− 0.027	1307	1686	− 0.127	10,828	79.72	3739	4893	− 0.134	1037	1159	− 0.056
*Rhipicephalus geigyi*	14,948	80.37	5886	6127	− 0.020	1293	1642	− 0.119	10,831	80.47	3828	4888	− 0.122	1023	1092	− 0.033
*Rhipicephalus microplus*	15,167	79.73	5888	6204	− 0.026	1376	1698	− 0.105	10,824	79.31	3711	4873	− 0.135	1074	1165	− 0.041
*Rhipicephalus sanguineus*	14,714	77.36	5545	5838	− 0.026	1478	1853	− 0.113	10,814	77.42	3641	4731	− 0.130	1119	1323	− 0.084
*Rhipicephalus turanicus*	14,717	77.81	5561	5890	− 0.029	1452	1814	− 0.111	10,811	77.88	3666	4754	− 0.129	1108	1283	− 0.073
*Rhipicentor nuttalli*	14,779	78.27	5581	5987	− 0.035	1380	1831	− 0.140	10,797	78.22	3598	4847	− 0.148	1090	1262	− 0.073
*Dermacentor everestianus*	15,191	78.80	5806	6165	− 0.030	1436	1784	− 0.108	10,520	78.33	3459	4781	− 0.160	1124	1151	− 0.012
*Dermacentor nitens*	14,839	77.42	5640	5849	− 0.018	1410	1940	− 0.158	10,520	77.16	3439	4678	− 0.153	1166	1237	− 0.030
*Dermacentor nuttalli*	15,086	78.93	5871	6036	− 0.014	1324	1855	− 0.167	10,877	78.80	3709	4862	− 0.135	1073	1223	− 0.065
*Dermacentor silvarum*	14,945	78.78	5812	5961	− 0.013	1336	1836	− 0.158	10,844	78.67	3680	4851	− 0.137	1077	1236	− 0.069
*Bothriocroton concolor*	14,809	75.14	5443	5685	− 0.022	1607	2704	− 0.254	10,910	74.44	3495	4626	− 0.139	1313	1476	− 0.058
*Bothriocroton undatum*	14,769	76.90	5464	5893	− 0.038	1540	1872	− 0.097	10,895	76.10	3546	4745	− 0.145	1237	1367	− 0.050
*Haemaphysalis bancrofti*	14,673	78.35	5687	5810	− 0.011	1381	1795	− 0.130	10,819	78.38	3712	4768	− 0.125	1137	1202	− 0.028
*Haemaphysalis concinna*	14,675	77.98	5665	5778	− 0.010	1350	1879	− 0.164	10,856	77.92	3692	4767	− 0.127	1129	1268	− 0.058
*Haemaphysalis flava*	14,689	76.88	5541	5752	− 0.019	1498	1898	− 0.118	10,824	76.62	3601	4692	− 0.132	1213	1318	− 0.041
*Haemaphysalis formosensis*	14,676	78.29	5667	5823	− 0.014	1369	1817	− 0.141	10,833	78.20	3703	4768	− 0.126	1130	1232	− 0.043
*Haemaphysalis hystricis*	14,716	77.22	5646	5718	− 0.006	1448	1904	− 0.136	10,820	76.77	3592	4714	− 0.135	1187	1327	− 0.056
*Haemaphysalis japonica*	14,685	77.58	5605	5788	− 0.016	1435	1845	− 0.125	10,833	77.60	3656	4750	− 0.130	1149	1278	− 0.053
*Haemaphysalis longicornis*	14,718	77.16	5618	5738	− 0.011	1440	1922	− 0.143	10,795	76.79	3595	4695	− 0.133	1190	1315	− 0.050
*Haemaphysalis parva*	14,846	78.82	5806	5896	− 0.008	1342	1802	− 0.146	10,822	78.76	3685	4838	− 0.135	1088	1211	− 0.054
*Hyalomma asiaticum*	14,720	78.18	5600	5908	− 0.027	1374	1838	− 0.144	10,913	78.04	3663	4853	− 0.140	1116	1281	− 0.069

Metazoan mt-genomes usually have a higher adenine–thymine (AT) base content [22, 32, 42]. Analysis of base usage in tick mt-genomes showed that the AT content ranged from 80.45% (*Amblyomma elaphense*) to 65.23% (*Ornithodoros savignyi*) with an average content of 75.51% (Table 2). The difference in base usage within the family is generally small [48, 49], but the largest difference in AT content between soft and hard ticks reached 15.22%. This phenomenon may be attributed to the lower AT content in *Ornithodoros* species, which is 71.65% on average and is considerably lower than the average AT content of ticks. It is possible that the difference in AT content is related to the size of the NCRs, the repeat sequences and the complexity of the gene structure [50–52]. Additionally, the different living environments and survival strategies of soft and hard ticks influence base usage [53].

The base skew of tick mt-genomes is unique. In general, AT-skew is positive and guanine–cytosine (GC) skew is negative in the metazoan mt-genomes [54, 55], whereas the AT-skew of soft and hard ticks is different. In soft ticks, the AT-skew is positive. In hard ticks, the positive AT-skew is only observed in *I. hexagonus* and *Ixodes uriae*, whereas in other hard ticks, the AT skew is negative. In both soft and hard ticks, the average AT-skew is 0.0504 and − 0.0187, respectively, and the average GC-skew is − 0.3532 and − 0.1701, respectively; notably the difference in AT-skew is smaller than that in GC-skew (Table 2).

### Protein-coding genes and codon usage

The PCGs in mt-genomes encode several subunits: NADH dehydrogenase subunit, cytochrome *c* oxidase subunit, ATPase subunit and cytochrome *b*, which are mainly involved in the oxidative phosphorylation of cells [56]. The average length of mitochondrial PCGs in soft and hard ticks is 10,866 and 10,819 bp, respectively (Table 2). The AT content in PCGs of the soft ticks (71.81%) and hard ticks (77.36%) is also lower than that in the complete mt-genome level. The lowest AT content in PCGs is in *Rhipicephalus geigyi* (63.59%) and the highest is in *Ornithodoros savignyi* (80.47%). The base skew in PCGs of ticks is negative, and the skewness characteristics are similar in both soft and hard ticks. No obvious differences have been observed in different genera of ticks, and the level of AT-skew is higher than that of the GC-skew. The mitochondrial PCGs are involved in oxidative phosphorylation and energy production; therefore, the structure is relatively conserved, and the difference in base usage is lower than that of the whole genome. In addition, the higher AT content of tick mt-genomes may be influenced by gene sequences, with there being only a 0.11–1.64% gap between the AT content of PCGs and the whole mt-genome (Table 2).

Similarly to insects, ticks usually adopt the “ATN”-type codon as the initial codon in PCGs [31–34, 57]. Other codons, including some special initiation codons, can be edited to conventional start codons during transcription [58–60], which may help reduce the gene spacer region and overlapping region and not affect the normal translation of proteins [61]. The termination codons of ticks are mainly TAA and TAG [31, 34] and sometimes use “T” or “TA”, which may be converted into a complete termination codon by polyadenylation after translation [62, 63].

### Transfer RNA and ribosomal RNA genes

The mitochondrial tRNA gene length in ticks ranges from 50 to 90 bp, and most tRNA genes have a complete cloverleaf structure, including four principal structures: amino acid acceptor (AA) arm; TΨC (T) arm; anticodon (AC) arm; and dihydrouridine (DHU) arm [64]. No DHU arm structure exists in *trnS1* of the tick mt-genomes; a similar phenomenon is also observed in insects [20, 65, 66]. The distance from the anti-codon to the CCA terminus is hence maintained through the inverted L structure, which helps complete the gene function [67]. Additionally, base mismatches frequently occur in the secondary structure of the tick tRNA genes [68, 69]. The mismatch types are mainly G-U, U-G and U-U, which are similar to those of other insects [62, 70]. These mismatches may be related to the evolutionary mutations and may not affect the function of tRNA genes due to being corrected later [71].

The mitochondrial rRNA genes display a complex functional structure with a relatively slow evolution rate; these have long been used as population genetics markers [72]. The tick mt-genomes contain two single copy *12S* and *16S* rRNA genes. In recent years, the mitochondrial *12S* and *16S* rRNA genes have been extensively used as genetic targets in phylogenetic research of ticks [27, 36, 73]. Due to gene rearrangement, the position of the rRNA genes shifts in ticks, whereas the gene order and the location in the N strand remain unchanged. Previous reports have shown that the average genetic distance of different tick taxa was still very slight even after tens of million years of evolution. Slow nucleotide variation in rRNA genes may be caused by strict structural and functional limitations [27]. Therefore, to this end, using combined PCGs and rRNA genes to reconstruct the phylogenetic relationships and resolve the controversial genealogy of soft ticks may be one of the best methods [19].

### Gene rearrangement

**Fig. 1:**
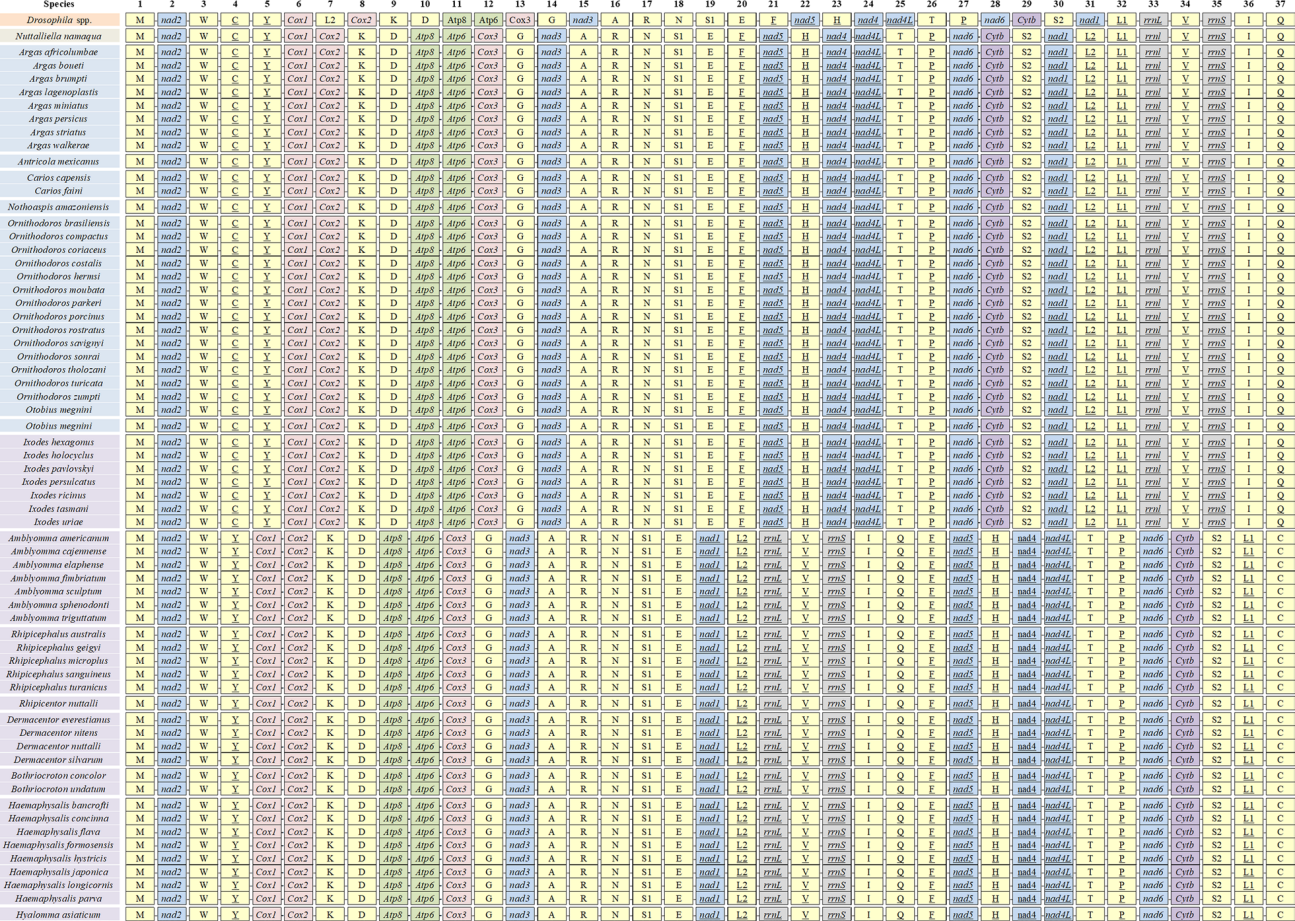
Gene rearrangement in the tick mitochondrial genomes

The mt-genomes exhibit higher rearrangement potential, but in general, the gene arrangement most likely occurs at a higher taxonomic level, which can provide insights for systematic classification at higher taxa [74, 75]. There are three types of changes in tRNA gene position: shuffling (local rearrangements), translocation (cross-gene displacement) and inversion (change in the encoding or transcriptional direction) [76]. The rearrangements in the tick mt-genomes are mainly divided into two patterns (Fig. 1). The arrangement of the soft ticks and *N. namaqua* show more similarity with that in the genus *Drosophila* [77, 78], which represents the ancestral arrangement in insects. In detail, shuffle (minor rearrangement of the gene) is observed only in the *trnL2* gene [48], which is moved from *cox*1–*cox*2 to *nad*1–*trnL1* with the coding strand changed from the J strand to the N strand, whereas other genes remain unchanged. In hard ticks, a major gene rearrangement is observed in a large gene region (*trnF-nad*5*-trnH-nad4–nad4L-trnT-trnP-cytb-trnS2*), which is moved from *trnE-nad*1 to *trnQ*-*trnM*. The major gene rearrangement involves the translocation of three tRNA genes (*trnL1*, *trnL2* and *trnC*) and the inversion of the *trnC* gene. The patterns in gene rearrangement might be associated with the rate of molecular evolution, and the different rearrangements between soft and hard ticks may have occurred from a very early period [74, 79].

### Non-coding regions

In insects, the transcription termination of the mitochondrial NCRs is realized by combining transcription termination factors [80]. In ticks, the mt-genome features a compact structure, which usually contains two conserved site-specific NCRs and several genus-specific conserved NCRs [19, 27, 28, 34, 39]. The larger NCR is located between *rrnS–trnI* and is approximately 200–400 bp long (Table 3). The length of NCR in soft and hard ticks averages 274 and 261 bp, respectively. The longest NCR is observed in species of the genus *Ixodes* with an average length of 336 bp. The shortest NCR is only 82 bp in *Rhipicentor nuttalli*, and the notably short NCR may be attributed to assembly errors. The other conservative NCRs are located between *rrnL* and *trnV*, and the length of this region varies greatly. The shortest is only 155 bp in *Amblyomma triguttatum*, and the longest reaches 565 bp in *Argas lagenoplastis*. The difference in the average length between the soft and hard ticks is only 1 bp (251 and 252 bp, respectively). The length difference of this type of NCR in ticks is often significant within a genus, except for the genus *Haemaphysalis*, which shares a similar length of 150 bp. In addition to the abovementioned two NCRs, there is another NCR located between *trnL1* and *trnC* in hard ticks. It is possible that the two related genes (*trnL1* and *trnC*) may be involved in gene rearrangement, and hence the NCRs may act as a fragment insertion and play specific roles during gene transcription [81, 82]. Additionally, some ticks also exhibit other NCRs, such as *Dermacentor nitens* and *A. triguttatum*, which display five NCRs. These NCRs may play important roles in protecting gene function during gene rearrangement, and there are currently four hypotheses to explain the formation of these particular NCRs [27, 33, 41, 74].

**Table 3:** Distribution of NCRs in the tick mitochondrial genomes

Species	Conservative noncoding region						Nonconservative noncoding region			
	Length	Position	Length	Position	Length	Position	Length	Position	Length	Position
*Nuttalliella namaqua*	182	*rrnL–trnV*	229	*rrnS–trnI*			361	*trnF-nad*5		
*Argas africolumbae*	185	*rrnL–trnV*	293	*rrnS–trnI*						
*Argas brumpti*	184	*rrnL–trnV*	280	*rrnS–trnI*						
*Argas boueti*	553	*rrnL–trnV*	279	*rrnS–trnI*						
*Argas lagenoplastis*	565	*rrnL–trnV*	238	*rrnS–trnI*						
*Argas miniatus*	178	*rrnL–trnV*	273	*rrnS–trnI*						
*Argas persicus*	179	*rrnL–trnV*	248	*rrnS–trnI*						
*Argas striatus*	182	*rrnL–trnV*	295	*rrnS–trnI*			112	*nad*2*-trnW*		
*Argas walkerae*	177	*rrnL–trnV*	272	*rrnS–trnI*						
*Antricola mexicanus*	189	*rrnL–trnV*	264	*rrnS–trnI*			104	*nad*2*-trnW*		
*Carios capensis*	177	*rrnL–trnV*	308	*rrnS–trnI*						
*Carios faini*	188	*rrnL–trnV*	259	*rrnS–trnI*						
*Nothoaspis amazoniensis*	186	*rrnL–trnV*	264	*rrnS–trnI*			124	*trnF-nad*5		
*Ornithodoros brasiliensis*	193	*rrnL–trnV*	294	*rrnS–trnI*						
*Ornithodoros compactus*	176	*rrnL–trnV*	267	*rrnS–trnI*						
*Ornithodoros coriaceus*	189	*rrnL–trnV*	283	*rrnS–trnI*						
*Ornithodoros costalis*	190	*rrnL–trnV*	254	*rrnS–trnI*						
*Ornithodoros hermsi*	188	*rrnL–trnV*	269	*rrnS–trnI*						
*Ornithodoros moubata*	176	*rrnL–trnV*	283	*rrnS–trnI*						
*Ornithodoros parkeri*	192	*rrnL–trnV*	257	*rrnS–trnI*						
*Ornithodoros porcinus*	174	*rrnL–trnV*	265	*rrnS–trnI*						
*Ornithodoros tratus*	190	*rrnL–trnV*	289	*rrnS–trnI*						
*Ornithodoros avignyi*	181	*rrnL–trnV*	266	*rrnS–trnI*			125	*trnF-nad5*		
*Ornithodoros sonrai*	563	*rrnL–trnV*	255	*rrnS–trnI*						
*Ornithodoros tholozani*	554	*rrnL–trnV*	292	*rrnS–trnI*						
*Ornithodoros turicata*	189	*rrnL–trnV*	286	*rrnS–trnI*			122	*nad4–nad4L*		
*Ornithodoros zumpti*	564	*rrnL–trnV*	271	*rrnS–trnI*						
*Otobius megnini*	195	*rrnL–trnV*	290	*rrnS–trnI*						
*Ixodes hexagonus*	189	*rrnL–trnV*	268	*rrnS–trnI*						
*Ixodes holocyclus*	335	*rrnL–trnV*	349	*rrnS–trnI*	335	*trnL1–trnC*				
*Ixodes pavlovskyi*	193	*rrnL–trnV*	351	*rrnS–trnI*						
*Ixodes persulcatus*	183	*rrnL–trnV*	282	*rrnS–trnI*			122	*trnH-nad*4		
*Ixodes ricinus*	197	*rrnL–trnV*	351	*rrnS–trnI*			107	*nad*2*-trnW*		
*Ixodes tasmani*	481	*rrnL–trnV*	366	*rrnS–trnI*			145	*nad4–nad4L*		
*Ixodes uriae*	354	*rrnL–trnV*	385	*rrnS–trnI*	354	*trnL1–trnC*				
*Amblyomma americanum*	169	*rrnL–trnV*	237	*rrnS–trnI*	306	*trnL1–trnC*				
*Amblyomma cajennense*	172	*rrnL–trnV*	283	*rrnS–trnI*	306	*trnL1–trnC*				
*Amblyomma elaphense*	515	*rrnL–trnV*	238	*rrnS–trnI*	299	*trnL1–trnC*	127	*nad2-trnW*		
*Amblyomma fimbriatum*	165	*rrnL–trnV*	230	*rrnS–trnI*	274	*trnL1–trnC*				
*Amblyomma sculptum*	172	*rrnL–trnV*	247	*rrnS–trnI*	306	*trnL1–trnC*				
*Amblyommas phenodonti*	158	*rrnL–trnV*	297	*rrnS–trnI*	328	*trnL1–trnC*				
*Amblyomma triguttatum*	155	*rrnL–trnV*	264	*rrnS–trnI*	307	*trnL1–trnC*	123	*nad2-trnW*	185	*trnF-nad*5
*Rhipicephalus australis*	157	*rrnL–trnV*	265	*rrnS–trnI*	305	*trnL1–trnC*				
*Rhipicephalus geigyi*	541	*rrnL–trnV*	244	*rrnS–trnI*	303	*trnL1–trnC*	241	*trnE-nad*1		
*Rhipicephalus microplus*	561	*rrnL–trnV*	264	*rrnS–trnI*	307	*trnL1–trnC*	124	*nad*2*-trnW*		
*Rhipicephalus sanguineus*	157	*rrnL–trnV*	233	*rrnS–trnI*	303	*trnL1–trnC*				
*Rhipicephalus turanicus*	159	*rrnL–trnV*	240	*rrnS–trnI*	304	*trnL1–trnC*				
*Rhipicentor nuttalli*	157	*rrnL–trnV*	82	*rrnS–trnI*	308	*trnL1–trnC*	285	*trnE-nad*1		
*Dermacentor everestianus*	569	*rrnL–trnV*	292	*rrnS–trnI*	306	*trnL1–trnC*	322	*trnE-nad*1	119	*trnQ-trnF*
*Dermacentor nitens*	556	*rrnL–trnV*	235	*rrnS–trnI*	307	*trnL1–trnC*	168	*trnE-nad*1	166	*trnQ-trnF*
*Dermacentor nuttalli*	556	*rrnL–trnV*	235	*rrnS–trnI*	307	*trnL1–trnC*	168	*trnE-nad*1		
*Dermacentor silvarum*	556	*rrnL–trnV*	232	*rrnS–trnI*	307	*trnL1–trnC*	167	*trnE-nad*1		
*Bothriocroton concolor*	162	*rrnL–trnV*	247	*rrnS–trnI*	311	*trnL1–trnC*				
*Bothriocroton undatum*	157	*rrnL–trnV*	230	*rrnS–trnI*	310	*trnL1–trnC*	113	*nad4–nad4L*		
*Haemaphysalis bancrofti*	163	*rrnL–trnV*	262	*rrnS–trnI*	307	*trnL1–trnC*				
*Haemaphysalis concinna*	161	*rrnL–trnV*	230	*rrnS–trnI*	311	*trnL1–trnC*				
*Haemaphysalis flava*	158	*rrnL–trnV*	228	*rrnS–trnI*	311	*trnL1–trnC*				
*Haemaphysalis formosensis*	160	*rrnL–trnV*	265	*rrnS–trnI*	311	*trnL1–trnC*				
*Haemaphysalis hystricis*	162	*rrnL–trnV*	228	*rrnS–trnI*	309	*trnL1–trnC*				
*Haemaphysalis japonica*	156	*rrnL–trnV*	229	*rrnS–trnI*	310	*trnL1–trnC*				
*Haemaphysalis longicornis*	159	*rrnL–trnV*	240	*rrnS–trnI*	309	*trnL1–trnC*				
*Haemaphysalis parva*	158	*rrnL–trnV*	252	*rrnS–trnI*	318	*trnL1–trnC*	211	*trnE-nad*1		
*Hyalomma asiaticum*	160	*rrnL–trnV*	287	*rrnS–trnI*	307	*trnL1–trnC*				

It is noteworthy that a common marker sequence is found in the NCRs of the tick mt-genomes, which are formed by degeneration during evolution and named the “Tick-box” [39]. This conserved sequence is located at the boundary of two gene rearrangement regions in the tick mt-genomes, which may be affected by the arrangement of mitochondrial genes in ticks [27, 36]. However, this sequence is not discarded during long-term evolution and likely functions as a transcriptional maturation or termination signal. Annotation of these sequences can help identify hidden molecular functions, which is useful for genetic analysis of higher taxa [39].

### Mt-genome phylogeny

The mt-genomes play an important role in the molecular systematics and origin of ticks. In the present study, 13 PCGs and 2 rRNA genes from the MITOS analysis results of all available tick complete mt-genomes were used to construct a phylogenetic tree through the maximum likelihood method (ML) [83]. MEGA v.6.0 for Windows (https://www.megasoftware.net/) was first used for alignment and splicing, and then the IQ-Tree online server (http://iqtree.cibiv.univie.ac.at/) was used for establishment of the phylogenetic tree with 1000 bootstrap replications [84, 85]. The phylogenetic tree was constructed using the nucleotide sequences (12,150 bp) of 63 tick species. *Limulus polyphemus* (NC003057) was used as the outgroup and the percentage of the bootstrap support is given at each node.

**Fig. 2:**
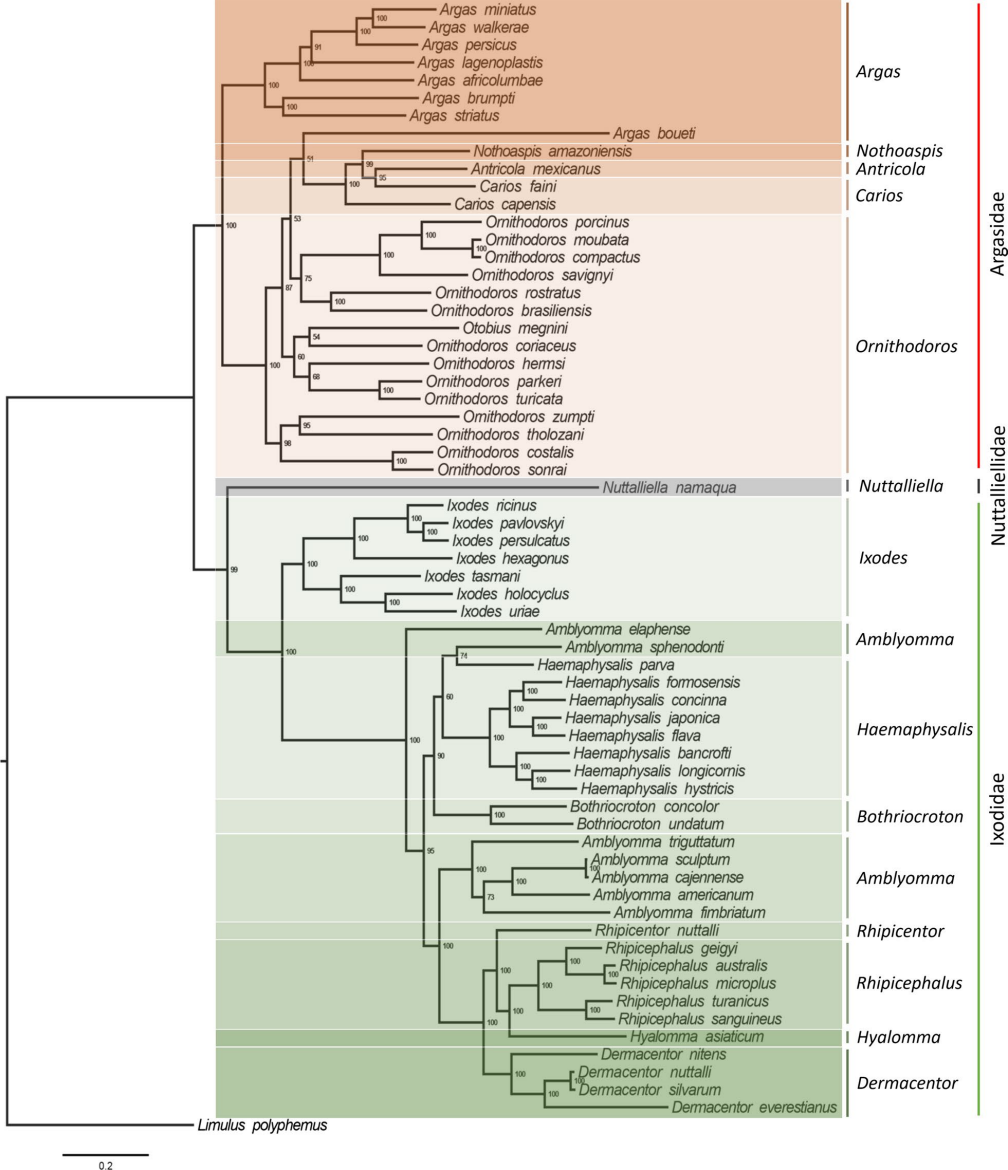
The phylogenetic tree shows the evolutionary relationships among tick species based on the complete mt-genome (13 PCGs and 2 rRNA). The tree was constructed using ML analysis of the 13 PCGs and 2 rRNA nucleotide sequences (12,150 bp) of 63 tick species. *Limulus polyphemus* (NC003057) is the outgroup. In the phylogenetic tree, the scale-bar represents the number of expected changes per site. Percentage of the bootstrap support is given at each node. The gray, red and green areas indicate species of Nuttalliellidae, Argasidae and Ixodidae, respectively. GenBank accession numbers are listed in Table 1

In soft ticks, some species in *Argas* and *Ornithodoros* have previously been phylogenetically analyzed using 10 mitochondrial genes [27]. Recently, several new mt-genomes have become available for the genus *Argas* including *Ar. boueti*, *Ar. brumpti*, *Ar. persicus*, *Ar. striatus* and *Ar. walkerae*, and for the genus *Ornithodoros* including *O. compactus*, *O. coriaceus*, *O. costalis*, *O. hermsi*, *O. parkeri*, *O. sonrai*, *O. tholozani*, *O. turicata* and *O. zumpti*. These were incorporated into the present phylogenetic analysis using 13 PCGs and 2 rRNA genes. Results yielded ambiguous species delimitation and phylogenetic relationships of these two genera (Fig. 2), which are complicated with the existing of monophyly, paraphyly, or polyphyly phenomena. Possibly, the concatenation of present genes with other informative genes help a better phylogenetic resolution. The tick *Ar. boueti* was clustered within the subfamily Ornithodorinae with a minimum bootstrap of 51%. This clustering may influence the location of other genera, including *Antricola*, *Nothoaspis* and *Carios*. Additionally, the tick *Carios faini* was clustered first with *Antricola mexicanus* and *Nothoaspis amazoniensis*, as well as with *C. capensis*. Subsequently, the incongruence was apparent between phylogenetic configurations and morphological characterizations, which requires further evidential confirmation.

In hard ticks, *Rhipicentor nuttalli* was clustered with species within the genus *Rhipicephalus*, which provided corroborative evidence for their close relationship. Although most clades among the hard ticks in different genera showed moderate support and the clustering of the tick lineages were similar to previous studies [25], some particular species including *Amblyomma elaphense*, *Am. spnenodonti* and *Hylomma asiaticum* require total evidence support. The only tick in the family Nuttalliellidae, *Nuttalliella namaqua*, is the sister group of the family Ixodidae, which is similar to the previous mt-genome phylogenetic analysis [27].

ML analysis of mitochondrial genes is widely used in the molecular systematics of ticks [19, 29, 34]. Although there were some changes in our results, the phylogenetic branching results were similar to those obtained based on ten PCGs [27]. This finding suggests that the combination of more mitochondrial genes may provide more robust evidence for tick taxonomy. Different mitochondrial genes or sites usually have different evolutionary rates, which may affect the topological structure and lower the support rate of the phylogenetic tree, thereby affecting the reliability of phylogenetic results [86, 87]. When the data matrix is partitioned according to both genes and coding sites, the phylogenetic calculation will be difficult to converge, which prevents phylogenetic analysis using a large number of mitochondrial genes simultaneously [88]. Thus, most studies usually adopt different PCGs or gene loci with proper partition, and the calculation can be optimized by modifying gene loci and selecting appropriate phylogenetic tree methods [89, 90]. Previous research based on morphological and nuclear rRNA data supported the cladistic results of Klompen et al. [19, 91]. The results obtained by combining multiple mitochondrial PCGs are partly different from those obtained using nuclear rRNA alone. Although some genera clades may change with the increasing number of mt-genomes, most genera remain clustered in the same clades [31–34] (Fig. 2). Molecular evidence based on the mt-genomes largely does not disagree with the recognized phylogenetic status of many tick species [12]. The description of new species and the characterization of new genetic markers will serve to systematically classify ticks [92].

### Perspectives and future directions

Ticks and mites of the subphylum Chelicerata account for 53% of parasitic arthropods, which cause substantial losses in agriculture and human health [93]. In recent years, the mt-genomes have shown significant advantages and have been widely used in taxonomic and phylogenetic research [19, 36, 94]. However, challenges still exist in systematic investigations on the tick mt-genomes. The number of available mt-genomes remains limited, as only 63 complete tick mt-genomes are presently available in the NCBI database; the complete mt-genomes of approximately 93% of tick species remain unexplored. The absence of complete tick mt-genomes, especially for some soft ticks with geographical and taxonomic bias will undoubtedly hinder the reliability of the cladistics (phylogenetic) of the species within subclass Acari, order Ixodida. The different evolution rates of mitochondrial genes may lead to variation in gene length of many species, and different sequences. It should be mentioned that the annotation methods would be also able to affect the sequence assembly [94, 95]. Furthermore, the mitochondrion is essential for energy metabolism and temperature regulation in metazoans [96]. Previous studies have shown that the mitochondrial genes have significantly different transcriptional activities during the freezing or anoxia adaptation and organism development [97–100]. The differential expression of specific functional genes may attribute to adaptive evolution [101]. Finally, no genes are encoded by the NCRs; therefore, NCRs receive less selection pressure during the process of evolution and are prone to base mutations [102]. NCRs can regulate gene expression and have many multiple tandem repeats and complex structures; hence, NCRs are more difficult to sequence [18, 102]. The tick mt-genomes are characterized by two typical conserved NCRs, but there are significant differences in the length, number, and location among the different species.

Due to the above challenges, several important directions for future research on the tick mt-genomes were prospected. First, more complete mt-genome sequences, combing with morphological characteristics and nucleus sequences, are required to integrately illuminate the phylogenetic relationships within Ixodida. Secondly, through extensive practices, mt-genome annotation methods are constantly improving [94]. However, annotation of a genome is still challenging, as different annotation methods may result in annotation bias or errors [102]. Hence, it is important to use unified annotation methods to help reduce or eliminate incorrect sequencing errors, and more attention should be given to NCRs. Thirdly, the functions and physiological relevance of the tick mitochondrial genes, including mitochondrial transcription, proteomics analysis of mitochondrial proteins, and epigenetic regulation in mitochondria under environmental or physiological stress, warrant further investigation. Finally, it is of considerable practical and theoretical interest to determine whether insecticides and acaricides can act on tick mitochondrial PCGs, which have been previously proved in mites [103, 104]. This knowledge may provide new molecular biology information to further understand the genetic diversity of ticks, and shed light on novel strategies to control TBDs damage.

## Conclusions

This study summarizes the basic features, including genomic structure, base difference and gene arrangement, of the tick mt-genomes available in the NCBI database. Research on tick mt-genomes has lagged behind that conducted in insects. Fortunately, an increasing number of mt-genomes have been published in recent years, and these have become important molecular markers for the phylogeny of ticks. Our study constructed a phylogenetic tree by maximum likelihood using 13 PCGs and 2 rRNA genes, and the results further supported the phylogenetic status of many tick species. Undoubtedly, the application of polygenic joint analysis and appropriate software will be widely applied in solving the phylogenetic and genetic evolution of diverse taxa of ticks, which will be of profound significance for the rapid identification of tick species.

## Data Availability

Not applicable.

## Abbreviations

TBDs: tick-borne diseases
SFTSV: severe fever with thrombocytopenia syndrome virus
TBEV: tick-borne encephalitis virus
ALSV: Alongshan virus
PCGs: protein-coding genes
tRNA: transfer RNA
rRNA: ribosomal RNA
NGS: next-generation sequencing
NCRs: non-coding regions
J strand: majority strand
N strand: minority strand
ML: maximum likelihood
